# A nasal omicron vaccine booster elicits potent neutralizing antibody response against emerging SARS-CoV-2 variants

**DOI:** 10.1080/22221751.2022.2053365

**Published:** 2022-03-30

**Authors:** Joy-Yan Lam, Yau-Yee Ng, Chun-Kit Yuen, Wan-Man Wong, Kwok-Yung Yuen, Kin-Hang Kok

**Affiliations:** a Department of Microbiology, Li Ka Shing Faculty of Medicine, The University of Hong Kong, Hong Kong, People’s Republic of China; b Centre for Virology, Vaccinology and Therapeutics, Hong Kong Science and Technology Park, Hong Kong SAR, People’s Republic of China; c State Key Laboratory of Emerging Infectious Diseases, The University of Hong Kong, Hong Kong, People’s Republic of China

**Keywords:** COVID-19, SARS-CoV-2, nasal protein vaccine booster, delta variant, omicron variant

## Abstract

SARS-CoV-2 has caused the COVID-19 pandemic since early 2020. As of January 2022, the worldwide spreading of SARS-CoV-2 leads to approximately 0.35 billion of human infections and five millions of deaths. Current vaccination is one of the effective ways to control SARS-CoV-2 transmission and reduce the disease severity. However, the antibody level against the immunogen significantly drops several months after the standard two-dose vaccination, and hence a third or fourth dose booster (the same immunogen) has been suggested to boost the antibody response. Here, we described an ultra-effective nasal vaccine booster that potently induced the extraordinary high-level of neutralizing antibody in pre-vaccinated mice. The vaccine booster is composed of a recombinant receptor binding domain of SARS-CoV-2 spike (either wild-type or omicron) fused with a domain of SARS-CoV-2 nucleoprotein. In the absence of adjuvants, a single intranasal administration of the booster in pre-vaccinated mice significantly induced systemic and mucosal antibody responses as evidenced by the elevation of the cross-variant neutralizing antibody and induction of IgA in bronchoalveolar lavage respectively. Most importantly, the single dose nasal vaccine booster (omicron version) potently enhanced the neutralizing activity against authentic SARS-CoV-2 omicron virus infection. Taken together, the induction of respiratory mucosal immunity and the enhancement of cross-variant neutralizing activity by the nasal vaccine booster warrants further clinical trials in humans.

## Main

As of 26 January 2022, the SARS-CoV-2 virus has caused for more than 350 million confirmed COVID-19 cases of infection worldwide. Vaccination remains the most crucial preventive measure against the disease. Currently, several types of COVID-19 vaccines have been widely employed, including inactivated whole virions, adenoviruses, protein subunits and mRNA vaccines. Two doses of intramuscular vaccination of either of the vaccines generally elicit high-level neutralizing antibodies (nAbs) that can effectively neutralize the original SARS-CoV-2 and the subsequently emerged variant strains (for examples alpha and beta variants), but to a lesser extent to delta variants [1,2]. However, the recently emerged omicron variant encodes a spike protein carrying more than 30 point mutations [3,4], some of which are located at the binding sites of neutralizing antibodies [5,6]. This is one of the key reasons accounted for the recent high incidence of breakthrough omicron infections [7,8]. In view of the compromised neutralization against the most recently emerged omicron variant, plus the significant decline of nAb level after 4–6 months of vaccination, a third dose of vaccination has been suggested and is already implemented in some countries. It is also predictable that frequent boosting of antibody response may be required to maintain the level of nAbs high enough to combat the future emerging variants.

**Figure 1.**
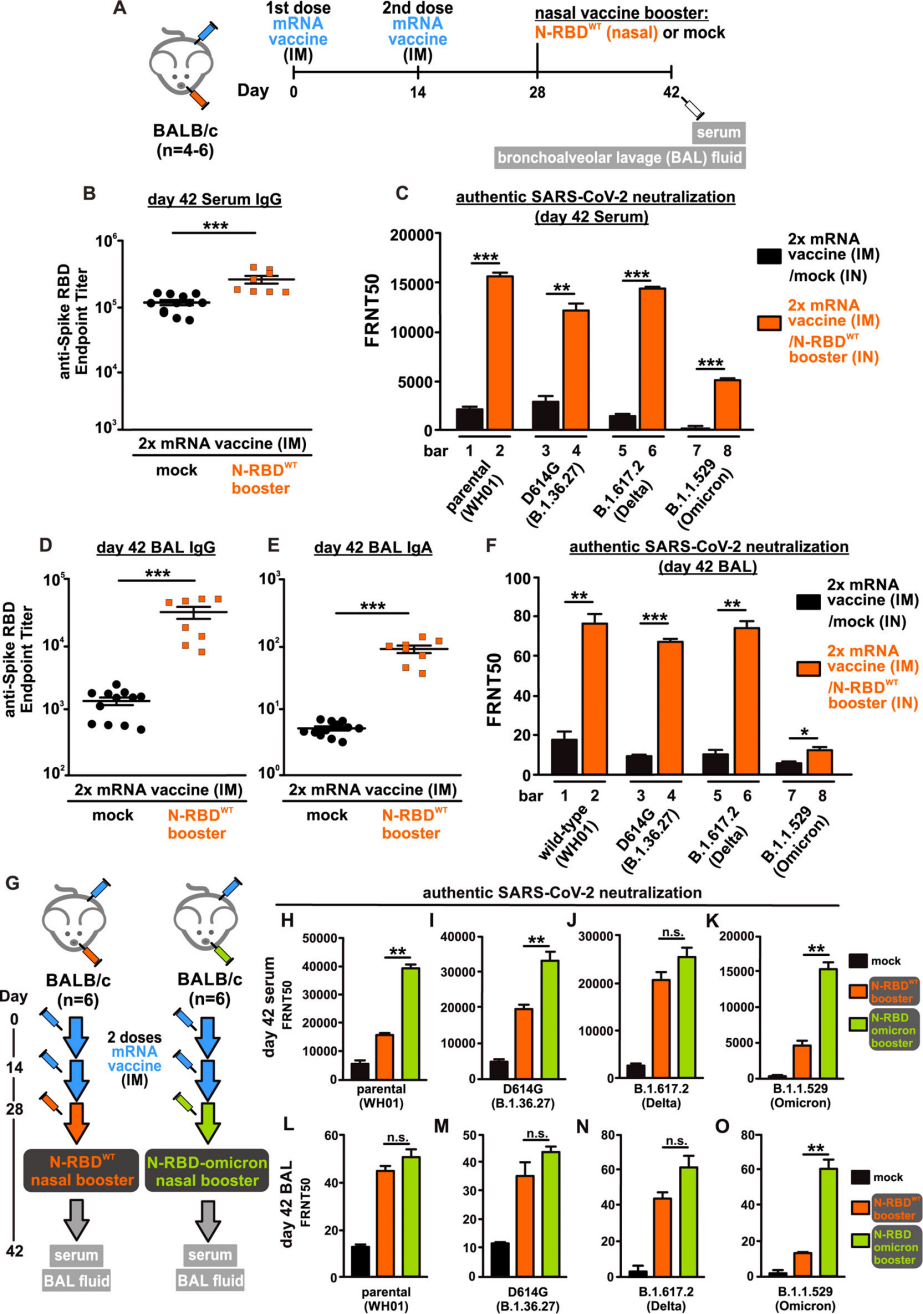
Neutralizing antibodies elicited by nasal N-RBD vaccine boosters. (A) Experimental design for nasal vaccine booster. Mice were intramuscularly injected with 2 doses of COVID-19 mRNA vaccine (1 µg per mouse), with 14 days apart. At day 28, 18 µg N-RBD^WT^ (recombinant SARS-CoV-2 spike RBD fused with nucleocapsid NTD) was administered intranasally for the booster group (n = 4 biological replicates). PBS was given intranasally for the control group (n = 6 biological replicates). Sera and bronchoalveolar lavage (BAL) fluids were collected at day 42. (B) Serum anti-SARS-CoV-2 spike RBD antibody at day 42 was detected by ELISA and presented as IgG endpoint titer. (C) SARS-CoV-2 virus neutralization of pooled sera from day 42 was quantitated by focus reduction neutralization (FRNT) assay. Data were shown as the reciprocal of dilution where 50% focus reduction was detected (FRNT_50_). (D and E) BAL anti-SARS-CoV-2 spike RBD IgG (D) and IgA (E) at day 42 was determined by ELISA. (F) SARS-CoV-2 virus neutralization of pooled BAL fluids from day 42 was quantitated by FRNT assay and presented as FRNT_50_. (G) Similar to A, mice were given 2 doses of COVID-19 mRNA vaccines, and N-RBD^WT^ or N-RBD^Omicron^ booster were administered intranasally at day 28 (n = 6 biological replicates). (H-K) SARS-CoV-2 virus neutralization of pooled sera obtained from N-RBD^WT^ and N-RBD^Omicron^ booster groups at day 42 were quantitated by FRNT assay. (L-O) Neutralization of pooled BAL fluids obtained from N-RBD^WT^ and N-RBD^Omicron^ booster groups at day 42. Samples were measured in duplicates and all data points (8-12 replicates) were included in statistical analysis. Statistical tests were performed using two-tailed unpaired t-test. (*: *p* < 0.1; **:*p* < 0.05; ***:*p* < 0.005; n.s.: not significant).

With respect to this, we designed and tested the novel idea of using a chimeric SARS-CoV-2 protein (N-RBD^WT^), which composes of spike receptor-binding domain (RBD) fused with a domain of nucleocapsid protein, as a nasal vaccine booster without adjuvant. A mouse vaccination model was designed to determine the booster efficacy (Figure 1(A)). Groups of mice first completed the primary vaccination by intramuscular injection of two doses of COVID-19 mRNA vaccine (BioNTech) with a time interval of fourteen days between the two injections. Fourteen days after the second dose, the nasal vaccine booster (18 µg per mouse) or PBS (20 µL) were administered intranasally to the mice. Sera and bronchoalveolar lavage (BAL) fluids were collected for the detection of RBD-specific antibodies and nAbs against parental SARS-CoV-2 and variants. As expected, sera of mice vaccinated with two doses of COVID-19 mRNA vaccine contained high level of anti-RBD IgG antibodies, the mean endpoint titer of which reached 1.1 × 10^5^. Surprisingly, the nasal booster could further boost the serum anti-RBD IgG level from 1.1 × 10^5^ to 2.6 × 10^5^ (Figure 1(B)). Next, we examined the serum neutralizing ability against authentic live viruses including wild-type SARS-CoV-2 (Wuhan-Hu-1; WH01), D614G (B.1.36.27), delta (B.1.617.2) and omicron (B.1.1.529) variants (Figure 1(C)). First, we showed that nasal booster greatly enhanced the authentic live virus neutralization against wild-type SARS-CoV-2 (Wuhan-Hu-1) from FRNT_50 _= 2.2 × 10^3^ to 15.6 × 10^3^ (bar 1 compared to bar 2, Figure 1(C)). It is worthy to note that, without the nasal booster, there were a 30% and 86% reduction in neutralization against delta and omicron variants respectively (black bar 1 compared to black bar 5 and 7, Figure 1(C)). In line with recent reports, neutralization against the omicron variant drastically dropped, which could account for the recently increasing number of breakthrough infections worldwide [9]. Surprisingly, a single dose of nasal N-RBD^WT^ booster not only potentiated the neutralization against authentic SARS-CoV-2 and delta variant but also the omicron variant (orange bar 6 and 8, Figure 1(C)). Importantly, the N-RBD^WT^ nasal booster strongly induced SARS-CoV-2-specific mucosal immunity as evidenced by the 21-fold increase in IgG and 11-fold increase in secreted IgA against RBD in BAL (Figure 1(D,E)). The booster also enhanced the degree of neutralization against wild-type, D614G, delta and omicron viruses in BAL fluids (Figure 1(F)). Taken together, both the elevated endpoint titer of anti-spike RBD antibodies in BAL and the enhanced neutralization against different variants of live SARS-CoV-2 viruses indicated successful induction of mucosal immunity. It is noteworthy that our nasal booster N-RBD^WT^ contains a nucleoprotein domain. We observed that a single dose of N-RBD^WT^ could also elicit N-specific antibody (supplementary Figure 1), implying that the nasal booster not only enhanced the preimmunized anti-spike RBD antibody response but also functioned as a new immunogen. Further study on the induction of lung resident memory B cells and follicular helper T cells by nasal booster will reveal the underlying mechanism on the induction of mucosal immunity after primary intramuscular vaccination.

Several reports clearly demonstrated that antibodies induced by the two-dose intramuscular injection of spike mRNA vaccine are less neutralizing against omicron variant [9,10]. It is of great interest to determine whether the replacement of wild-type RBD of our N-RBD^WT^ booster by omicron RBD could further increase the neutralization activity against the omicron variant without compromising the augmentation of neutralization against wild-type SARS-CoV-2 and delta variant. We repeated the immunization experiment in a mouse model and compared the neutralization activity induced by the booster of N-RBD^WT^ and N-RBD^Omicron^. Serum and BAL fluid were obtained at 42 days post-immunization for authentic virus neutralization assay (illustrated in Figure 1(G)). As expected, serum antibodies elicited by two doses of mRNA vaccine could potently neutralize the parental SARS-CoV-2 infection only (FRNT_50 _= 6 × 10^3^, black bar in Figure 1(H)), but not the omicron infection (FRNT_50 _= 5 × 10^2^, black bar in Figure 1(K)). Both N-RBD^WT^ and N-RBD^Omicron^ booster potently enhanced the neutralizing activity against wild-type, D614G, delta and omicron virus infection (orange and green bars in Figure 1(H–K)). Most importantly, N-RBD^Omicron^ potently enhanced the neutralizing activity against authentic omicron virus infection (green bars in Figure 1(K,O)). The booster effect elicited by single dose of N-RBD^Omicron^ was unprecedently effective, reaching FRNT_50 _= 1.5×10^4^. Since the omicron variant predominantly infects the upper respiratory tract and displays high transmissibility, further development of N-RBD^Omicron^ into human nasal vaccine booster could help to control the spreading of the circulating variants including omicron.

## Discussion

The use of recombinant proteins for vaccination has previously been explored. By applying the protein vaccine intranasally, it is possible to induce mucosal immunity to combat infections of the mucosa theoretically. Nonetheless, recent studies demonstrated that nasal protein vaccines could induce adaptive immune response only in the presence of strong adjuvants such as Alum and STING-agonist [11–13], indicating that nasal vaccination with recombinant proteins may not be a good approach to primary immunization. However, in this study, we designed a nasal COVID-19 protein vaccine that served as a booster after the standard two-dose intramuscular mRNA vaccination. In the absence of adjuvants, the recombinant protein alone was enough to boost the serum neutralizing antibody level against multiple SARS-CoV-2 variants, including the omicron variant. Importantly, antigen-specific IgA was detected in the bronchoalveolar lavage of immunized mice, indicating the successful induction of mucosal immunity by the nasal protein booster. We postulate that a strong primary immunization could be a key to the success of the subsequent boosting with our nasal protein vaccine, although the exact molecular mechanism awaits discovery. The fact that the production of recombinant protein is easily scalable and does not involve handling of infectious materials suggests that the nasal protein vaccine could be a cost-effective booster with minimal safety concerns. The absence of adjuvants also minimizes the chance of allergy or irritation to the mucosa. Together with its high potency in inducing cross-variant antibody response and mucosal immunity, nasal protein boosters warrant further studies and clinal trials in humans to combat the COVID-19 pandemic.

## Supplementary Material

Supplemental Material

## References

[1] Buchan SA, Chung H, Brown KA, et al. Effectiveness of COVID-19 vaccines against Omicron or Delta symptomatic infection and severe outcomes. medRxiv. 2022: 2021.12.30.21268565. 10.1001/jamanetworkopen.2022.32760 PMC9500552 36136332

[2] Bernal J L, Andrews N, Gower C, et al. Effectiveness of Covid-19 Vaccines against the B.1.617.2 (Delta) variant. N Engl J Med. 2021 Aug 12;385(7):585–594. 34289274 10.1056/NEJMoa2108891 PMC8314739

[3] Callaway E. Heavily mutated Omicron variant puts scientists on alert. Nature. 2021 Dec;600(7887):21. 34824381 10.1038/d41586-021-03552-w

[4] National Center for Immunization and Respiratory Diseases (NCIRD) DoVD. Science Brief: Omicron (B.1.1.529) Variant. Centers for Disease Control and Prevention2021. Available from: https://www.cdc.gov/coronavirus/2019-ncov/science/science-briefs/scientific-brief-omicron-variant.html. 34932278

[5] Dejnirattisai W, Huo J, Zhou D, et al. SARS-CoV-2 Omicron-B.1.1.529 leads to widespread escape from neutralizing antibody responses. Cell. 2022 Feb 3;185(3):467-484 e15. 10.1016/j.cell.2021.12.046 PMC8723827 35081335

[6] Flemming A. Omicron, the great escape artist. Nat Rev Immunol. 2022 Feb;22(2):75. 10.1038/s41577-022-00676-6 PMC8749340 35017722

[7] Israel A, Shenhar Y, Green I, et al. Large-scale study of antibody titer decay following BNT162b2 mRNA vaccine or SARS-CoV-2 infection. Vaccines (Basel). 2021 Dec 31;10(1):64. 10.3390/vaccines10010064 PMC8781423 35062724

[8] Khoury J, Najjar-Debbiny R, Hanna A, et al. COVID-19 vaccine – long term immune decline and breakthrough infections. Vaccine. 2021 Nov 26;39(48):6984–6989. 34763949 10.1016/j.vaccine.2021.10.038 PMC8556595

[9] Schmidt F, Muecksch F, Weisblum Y, et al. Plasma neutralization of the SARS-CoV-2 Omicron variant. N Engl J Med. 2021 Dec 30. 10.1056/NEJMc2119641 PMC8757565 35030645

[10] Muik A, Lui BG, Wallisch AK, et al. Neutralization of SARS-CoV-2 Omicron by BNT162b2 mRNA vaccine-elicited human sera. Science. 2022 Jan 18;375(6581):678-680. 10.1126/science.abn7591 PMC9836206 35040667

[11] An X, Martinez-Paniagua M, Rezvan A, et al. Single-dose intranasal vaccination elicits systemic and mucosal immunity against SARS-CoV-2. iScience. 2021 Sep 24;24(9):103037. 34462731 10.1016/j.isci.2021.103037 PMC8388188

[12] Du Y, Xu Y, Feng J, et al. Intranasal administration of a recombinant RBD vaccine induced protective immunity against SARS-CoV-2 in mouse. Vaccine. 2021 Apr 15;39(16):2280–2287. 33731271 10.1016/j.vaccine.2021.03.006 PMC7934688

[13] Jangra S, Landers JJ, Rathnasinghe R, et al. A combination adjuvant for the induction of potent antiviral immune responses for a recombinant SARS-CoV-2 protein vaccine. Front Immunol. 2021;12:729189. 34603303 10.3389/fimmu.2021.729189 PMC8481386

