# Supplementary material for: A nasal omicron vaccine booster elicits potent neutralizing antibody response against emerging SARS-CoV-2 variants
Source: Emerg Microbes Infect. 2022 Mar 30;11(1):964–7. doi: 10.1080/22221751.2022.2053365 (PMC8973333; doi:10.1080/22221751.2022.2053365)
Supplement: Supplemental Material [file TEMI_A_2053365_SM6954.zip › Suppl files/Supplementary Figure 2.docx]

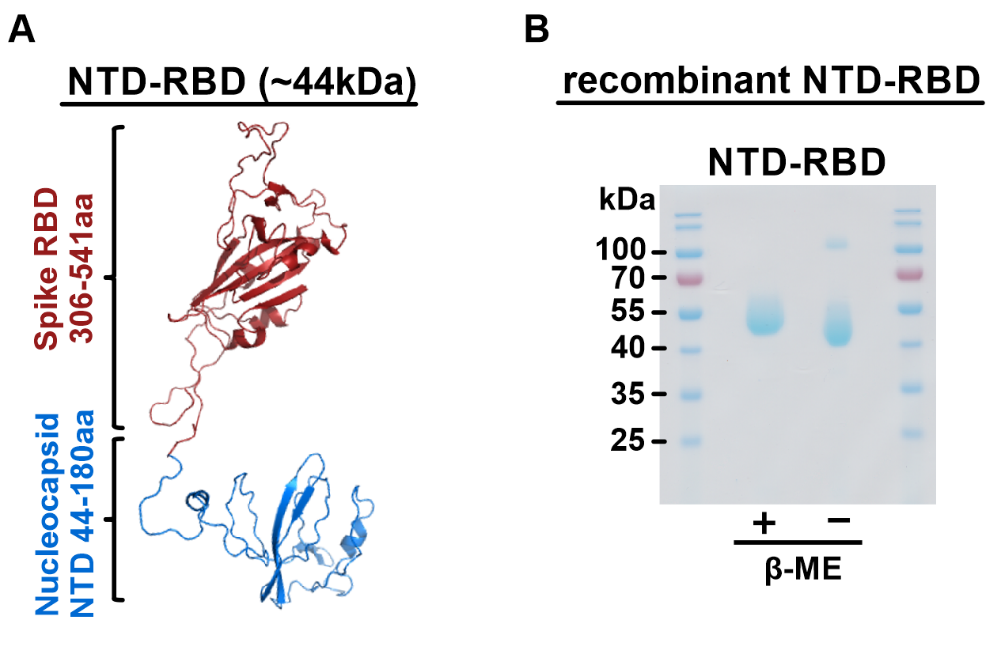


Supplementary Figure 2. N-RBD recombinant protein. SARS-CoV-2 nucleocapsid protein NTD (44-180aa) was fused with spike protein RBD (306-541aa). Recombinant protein was expressed in Expi293F cells and purified using Ni Sepharose Excel. **(A)** N-RBD protein structure prediction was performed using Robetta web server (https://robetta.bakerlab.org/). **(B)** SDS-PAGE gel staining by Coomassie G-250. N-RBD sample was analyzed with or without the reducing agent β-mercaptoethanol (β-ME).
