# Supplementary material for: A nasal omicron vaccine booster elicits potent neutralizing antibody response against emerging SARS-CoV-2 variants
Source: Emerg Microbes Infect. 2022 Mar 30;11(1):964–7. doi: 10.1080/22221751.2022.2053365 (PMC8973333; doi:10.1080/22221751.2022.2053365)
Supplement: Supplemental Material [file TEMI_A_2053365_SM6954.zip › Suppl files/Supplementary information_Methods and materials.docx]

Supplementary material 1

**Methods and materials**

**Protein expression and purification of N-RBD^WT^ and N-RBD^Omicron^**

Recombinant proteins were expressed using Expi293F expression system (Gibco). Gene sequences were codon-optimized and cloned into expression plasmids. For the N-RBD nasal booster, SARS-CoV-2 nucleocapsid protein (44-180aa) and spike RBD (306-541aa) were fused together by PCR and cloned into expression construct with a N-terminal 6xHis tag. To express mammalian recombinant proteins, plasmids constructs were transfected into Expi293F cells using Expifectamine 293 transfection reagent (Gibco) following manufacturer’s instruction and incubated for 72-96 hours. For protein purification, culture supernatant was harvested by centrifugation followed by filtration using 0.22µm filters (Millipore) and passed through Ni Sepharose Excel resin (Cytiva). The resin was then washed, and proteins were eventually eluted with buffer containing 160mM imidazole. Recombinant proteins were concentrated, and buffer exchanged using Pierce Protein Concentrator (Thermo).

**Animal experiment and vaccination**

Female 7-weeks old BALB/c mice were immunized intramuscularly with two doses (14 days in between) of COVID-19 mRNA vaccine (BioNTech) under anesthesia. Each dose comprised of 1 µg of mRNA vaccine per mouse. N-RBD protein boosters were dissolved in PBS/10% glycerol and administered intranasally (20µL) to the mice left nostril under anesthesia at a dose of 18µg protein per mouse. Control group was given PBS intranasally after two doses of intramuscular mRNA vaccine. Blood was collected from face vein at indicated timepoints and was centrifuged for serum collection. Animals were sacrificed by an overdose of anesthesia at the experiment endpoint. Bronchoalveolar lavage fluid was collected by inserting a catheter in the trachea of the terminally anesthetized mice, followed by instilling PBS into the bronchioles and lung. The collected fluid was centrifuged, and the supernatant was collected for downstream analysis. Animal experiments were approved by the Committee on the Use of Live Animals in Teaching and Research of the University of Hong Kong.

**Enzyme-Linked ImmunoSorbent Assay (ELISA)**

Antibody titers were determined by in-house established enzyme-linked immunosorbent assay (ELISA). High-binding 96-well microplates (Corning 3690) were coated with 5µg/mL recombinant SARS-CoV-2 spike RBD (306-541aa) protein with sodium carbonate buffer at 4°C for overnight. The next day, microplates were washed, blocked, and serially diluted samples were added. Bound antibodies were detected by HRP-conjugated secondary antibodies to mouse IgG (Abcam) or mouse IgA (Invitrogen) and 1-Step Ultra TMB-ELISA Substrate Solution (Thermo). Absorbance at 450nm was measured using Vatioskan LUX multimode microplate reader (Thermo) with SkanIt Software version 6.1.0.51. Titers were calculated in GraphPad Prism 8 by performing 4-parmeter logistical fitting on the absorbance data at each dilution. Antibody endpoint titers were determined as the interpolated reciprocal of the dilution giving the absorbance equivalent to the mean of control wells plus two standard deviations.

**Cell culture and viruses**

VeroE6 cells were cultured in DMEM (Gibco) supplemented with 10% fetal bovine serum (FBS) (Gibco). Viruses were isolated from clinical samples and cultured in BSL-3 laboratory. The accession number of representative SARS-CoV strains used in this study are MT230904 (WH01), MW856793 (B.1.36/D614G), OM212471 (B.1.617.2/Delta), and OM212472 (B.1.1.529/Omicron). Detailed information of the viruses was listed in supplementary table 1.

**Authentic SARS-CoV-2 virus neutralization**

Virus neutralization was quantitatively determined by in-house focus reduction neutralization assay and performed in BSL-3 laboratory. Briefly, VeroE6 cells were seeded in 96-well black plates (SPL) the day before the assay. On the day of assay, samples were serially diluted and incubated with 100-300 focus forming units (FFUs) of authentic live SARS-CoV-2 virus (WH01, B.1.36/D614G, B.1.617.2/Delta, B.1.1.529/Omicron) for 1 hour at 37°C. Sample-virus mixes were then transferred to VeroE6 cells for 1 hour at 37°C for inoculation. Cells were washed with PBS and replenished with culture medium containing 1% FBS. Inoculated cells were further incubated for 6 hours. At the assay endpoint, cells were fixed with 4% formalin. Fixed cells were washed with PBS, permeabilized with 0.1% NP40, blocked with 2% BSA, and stained with in-house rabbit anti-SARS-CoV-2 nucleocapsid protein polyclonal antibody and detected by anti-rabbit Alexaflour 488 (Abcam). Fluorescent positive foci were detected by Cytation 7 Cell Imaging Multi-Mode Reader (Biotek) with Gen5 Image Prime version 3.11.19. Neutralization titers were calculated and determined in GraphPad Prism 8 by performing 4-parameter logistical fitting on the detected fluorescent loci at each dilution. The 50% focus reduction neutralization titer (FRNT50) was determined as the interpolated reciprocal of the dilution having 50% reduction of infected fluorescent loci compared to control wells.
