# Supplementary material for: A nasal omicron vaccine booster elicits potent neutralizing antibody response against emerging SARS-CoV-2 variants
Source: Emerg Microbes Infect. 2022 Mar 30;11(1):964–7. doi: 10.1080/22221751.2022.2053365 (PMC8973333; doi:10.1080/22221751.2022.2053365)
Supplement: Supplemental Material [file TEMI_A_2053365_SM6954.zip › Suppl files/Suppplementary Figure 1.docx]

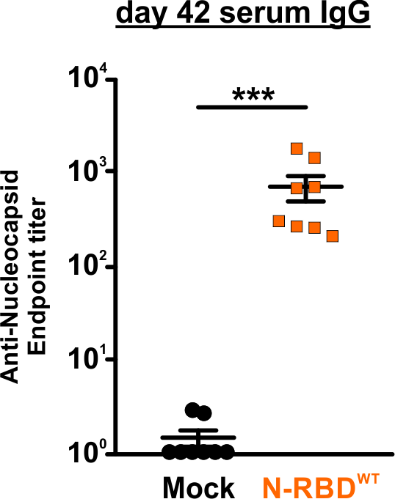


Supplementary Figure 1. Anti-nucleocapsid IgG elicited by N-RBD protein nasal vaccine booster. In house ELISA was used to detect nucleocapsid specific IgG in mice serum at day 42 (14 days after nasal vaccine booster). Microplates were coated with recombinant SARS-CoV-2 nucleocapsid protein. (***: p<0.005)
